# Targeted therapeutics and U.S. population-level mortality trends in multiple myeloma: A SEER-based analysis from 1975 to 2023

**DOI:** 10.18632/oncotarget.28877

**Published:** 2026-04-28

**Authors:** Navkirat Kahlon, Nahush Bansal, Sujatha Baddam, Jaisingh Rajput, Zaheer Qureshi

**Affiliations:** ^1^Mass General Cancer Center at Wentworth-Douglass Hospital, Dover, NH 03820, USA; ^2^University of Toledo Medical Center, Toledo, OH 43614, USA; ^3^Huntsville Hospital, Huntsville, AL 35801, USA; ^4^Montgomery Baptist Family Medicine Residency Program, Montgomery, AL 36116, USA; ^5^The Frank H. Netter M.D. School of Medicine at Quinnipiac University, Bridgeport, CT 06473, USA

**Keywords:** multiple myeloma, epidemiologic trends, mortality reduction, therapeutic advancements, SEER database

## Abstract

Multiple myeloma (MM), the second most common hematologic malignancy in the United States, has undergone transformative therapeutic innovation over the past five decades. Using SEER data from 1975 to 2023, we conducted a retrospective cross-sectional analysis to evaluate MM-specific mortality trends in relation to major treatment milestones. Age-adjusted mortality rates and Annual Percent Change (APC) were estimated using Joinpoint regression. Mortality increased from 1975 to 1994 (APC: 1.43%; *P* < .01), declined modestly from 1994 to 2002 (–0.70%; *P* = .02), dropped steeply from 2002 to 2009 (–1.85%; *P* < .01), plateaued between 2009 and 2014 (0.52%; *P* = .10), resumed decline from 2014 to 2021 (–1.73%; *P* < .01), and sharply decreased from 2021 to 2023 (–5.64%; *P* < .01). These inflection points align with the introduction of stem cell transplantation, proteasome inhibitors, immunomodulatory drugs, and next-generation immunotherapies including CAR T-cell therapy and bispecific antibodies. While these advances have improved survival, they also introduced chronic treatment burdens and rising costs. Our findings highlight the real-world impact of targeted therapies on population-level outcomes and underscore the urgent need for care models that ensure accessibility, affordability, and long-term sustainability in the era of precision oncology.

## INTRODUCTION

Multiple myeloma (MM) accounts for approximately 1% of global cancer-related mortality, with its incidence rising by 126% between 1990 and 2016 [1]. Historically, treatment options were limited to alkylating agents, corticosteroids, and combination regimens that offered modest survival benefit. The introduction of autologous stem cell transplantation (ASCT) in the mid-1990s marked a turning point, improving event-free survival compared to conventional chemotherapy [2].

Over subsequent decades, MM management has evolved through the development of targeted therapies, including proteasome inhibitors (PIs), immunomodulatory drugs (IMiDs), monoclonal antibodies (MAbs), selective inhibitors of nuclear export (SINEs), and more recently, cellular therapies such as CAR T-cells and bispecific antibodies. These agents act through diverse mechanisms—inducing apoptosis, modulating immune responses, and enhancing cytotoxicity [3–5]. Treatment intensification strategies, such as triplet regimens and maintenance therapy, have further contributed to improved outcomes [6].

The cumulative effect of these innovations is reflected in survival trends. The five-year relative survival rate for MM patients in the U.S. increased from 25% in 1975–1977 to 49% in 2005–2011 [7]. However, MM remains incurable and requires chronic therapy, often at escalating cost. This study analyzes MM-specific mortality trends using SEER data from 1975 to 2023, evaluating how evolving treatment strategies have shaped population-level outcomes over time.

## RESULTS

The age-adjusted U.S. mortality rate for multiple myeloma (MM) from 1975 to 2023 demonstrated dynamic shifts over time. These changes appear temporally aligned with evolving treatment strategies and broader improvements in supportive care, though causality cannot be inferred from population-level data alone.

### 1975–1994

MM mortality increased steadily during this period (APC: +1.43%; 95% CI: 1.33–1.55; *P* < .01), consistent with the limited efficacy of early regimens such as alkylating agents and corticosteroids.

### 1994–2002

A modest decline in MM-specific mortality was observed (APC: –0.70%; 95% CI: –1.03 to –0.21; *P* = .02), coinciding with the clinical adoption of autologous stem cell transplantation (ASCT).

### 2002–2009

A more substantial decline followed (APC: –1.85%; 95% CI: –2.78 to –1.50; *P* < .01), during a period marked by the introduction of immunomodulatory drugs (IMiDs) and proteasome inhibitors (PIs).

### 2009–2014

Mortality trends plateaued (APC: +0.52%; 95% CI: –0.10 to +1.69; *P* = .10). This interval saw refinements of existing drug classes, including next-generation IMiDs and PIs.

### 2014–2021

Mortality resumed a gradual decline (APC: –1.73%; 95% CI: –2.08 to –1.52; *P* < .01), overlapping with approvals for monoclonal antibodies, maintenance therapies, and optimized triplet combinations.

### 2021–2023

The sharpest decline was observed during this period (APC: –5.64%; 95% CI: –6.12 to –5.17; *P* < .01). This trend likely reflects both the impact of newly introduced agents—such as CAR T-cell therapies (e.g., ide-cel, cilta-cel) and bispecific antibodies (e.g., teclistamab)—and the cumulative benefits of prior therapeutic advances.

**Figure 1:**
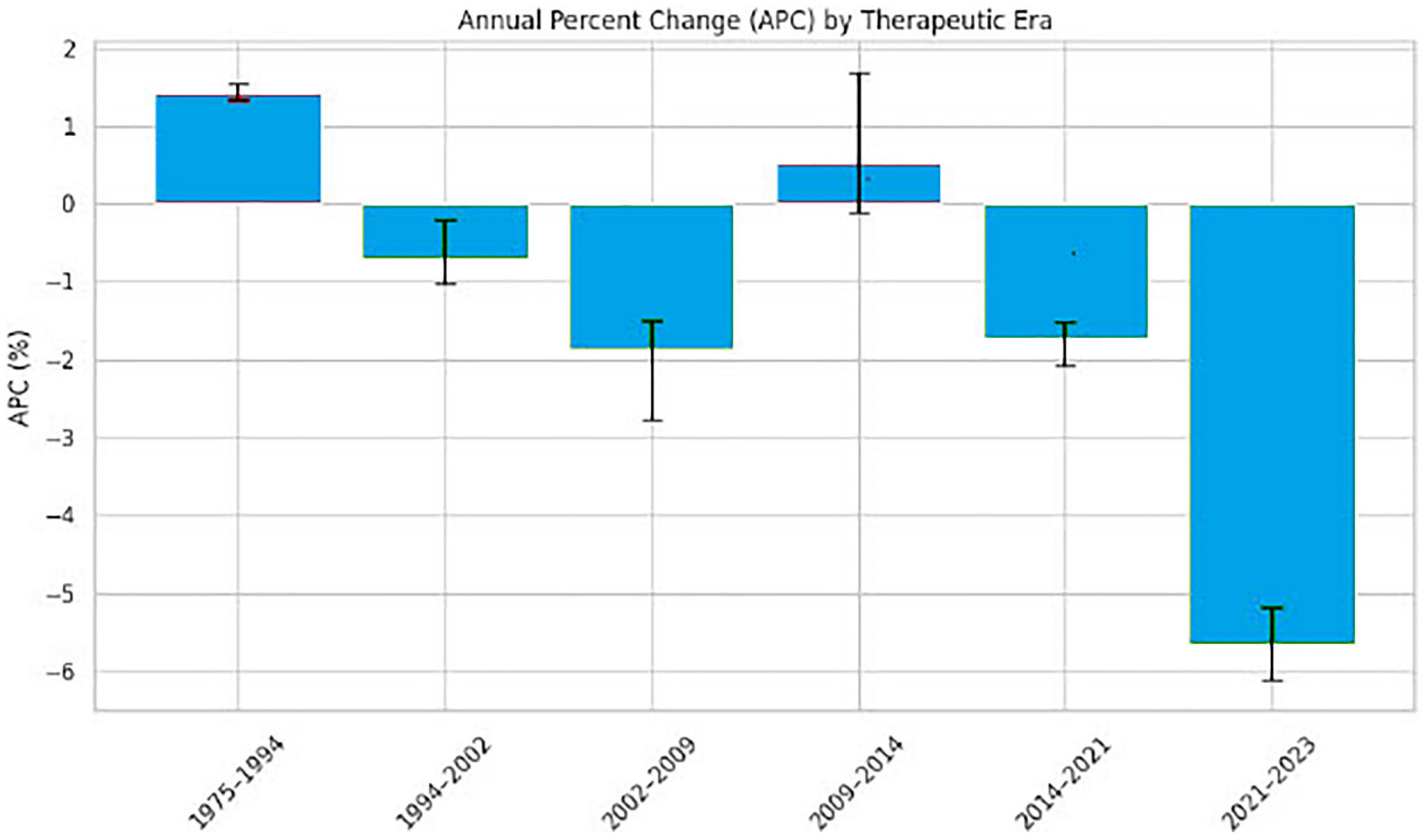
Annual percent change (APC) in multiple myeloma–specific mortality across six therapeutic eras in the United States, 1975–2023. Bars represent APC values derived from Joinpoint regression analysis using SEER data, with vertical lines indicating 95% confidence intervals. All bars are uniformly colored blue for visual consistency.

These results are summarized in Table 1 and Figure 1.

**Table 1:** Long-term trends in U.S. age-adjusted mortality rates, 1975–2023 and key developments

Calendar years	Age adjusted U.S. myeloma mortality rate; APC % (95% CI)	*P*-value	Trend	Key developments
**1975–1994**	+1.43 (1.33–1.55)	<.01	Increasing	Pre-transplantation era; melphalan and corticosteroids were standard with limited efficacy and minimal survival improvement
**1994–2002**	–0.70 (–1.03 to –0.21)	0.02	Modestly decreasing	Introduction of ASCT following high-dose melphalan—first major advancement improving survival
**2002–2009**	–1.85 (–2.78 to –1.50)	<.01	Steep decline	Incorporation of IMiDs (thalidomide, lenalidomide) and PIs (bortezomib)—first therapies showing PFS and OS benefits
**2009–2014**	+0.52 (–0.10 to +1.69)	0.10	No significant change	Plateau phase; refinement of existing drug classes like carfilzomib and pomalidomide; no novel class approvals
**2014–2021**	–1.73 (–2.08 to –1.52)	<.01	Gradual decline	Approval of monoclonal antibodies (daratumumab, elotuzumab), SINEs, and maintenance regimens; triplet combinations expand

## DISCUSSION

This population-based analysis of U.S. multiple myeloma (MM)-specific mortality from 1975 to 2023 highlights a sustained improvement in outcomes that coincides with the gradual evolution of therapeutic strategies. While causality cannot be inferred from aggregate data, the observed declines in mortality appear temporally aligned with successive treatment innovations—including autologous stem cell transplantation, immunomodulatory agents, proteasome inhibitors, monoclonal antibodies, and more recently, CAR T-cell therapies and bispecific antibodies. These advances have extended progression-free and overall survival in clinical settings and likely contributed to broader reductions in MM-specific mortality. Collectively, they have reshaped the epidemiology of MM, transitioning it from a rapidly fatal malignancy to a chronic, relapsing disease increasingly managed across multiple lines of therapy.

The age-adjusted U.S. mortality rate for multiple myeloma (MM) showed a sustained increase from 1975 to 1994, coinciding with a period of limited therapeutic efficacy. The primary regimen—melphalan, introduced in the 1950s and later combined with corticosteroids—was widely used but offered only marginal clinical benefit. Despite its ubiquity, this approach did not yield meaningful improvements in overall survival over more than three decades [8].

A pivotal shift in MM management occurred in the 1990s with the introduction of high-dose melphalan (HDM) followed by autologous stem cell transplantation (ASCT). ASCT was incorporated into frontline therapy to mitigate the profound myelosuppression associated with HDM, enabling dose intensification without prohibitive toxicity. This strategy demonstrated significant clinical benefit, with survival rates of 52% in the HDM plus ASCT group compared to 12% in the conventional therapy arm (*P* = 0.03) [2]. In parallel, MM-specific mortality rates began to decline between 1994 and 2002—marking the first sustained reversal in national death rates since 1974.

A more substantial decline in MM-specific mortality was observed between 2002 and 2009. This period coincided with the clinical introduction of two novel therapeutic classes—immunomodulatory drugs (IMiDs) and proteasome inhibitors (PIs)—which marked a transformative shift in MM management. The E1A00 trial established the efficacy of thalidomide, the first IMiD, demonstrating a median progression-free survival (PFS) of 22.6 months with thalidomide plus dexamethasone versus 6.5 months with dexamethasone alone [9, 10]. Although a clear overall survival (OS) benefit was not observed at that time, these results were unprecedented and underscored the therapeutic potential of IMiDs [11].

The advent of bortezomib, the first-in-class PI, represented another major milestone. In the VISTA trial, bortezomib combined with melphalan and prednisone yielded a median duration of response of 19.9 months compared to 13.1 months in the control group. Importantly, a significant OS benefit was observed, with a hazard ratio of 0.61 (*P* = .008), reinforcing its clinical impact [12, 13]. The decline in MM-specific mortality during this interval appears temporally aligned with the uptake of IMiDs and PIs—the first agents to demonstrate improvements in both PFS and OS—suggesting their contribution to improved population-level outcomes.

Between 2009 and 2014, no new therapeutic classes were introduced, and MM-specific mortality trends remained relatively stable. However, this period saw important refinements within existing drug categories. Carfilzomib, a next-generation PI, received FDA approval in 2012, and pomalidomide, a third-generation IMiD, was approved in 2013 for relapsed/refractory MM. Both agents demonstrated clinical efficacy in improving disease control [14, 15], expanding treatment options for patients with resistant disease.

From 2014 to 2021, a continued decline in MM-specific mortality was observed, marking a substantial improvement compared to the preceding four decades. This trend coincided with the cumulative introduction of novel agents, expanded combination regimens, and the formalization of maintenance therapy. In 2015, the FDA approved three distinct drug combinations for relapsed/refractory MM: panobinostat (a histone deacetylase inhibitor) with bortezomib and dexamethasone, ixazomib (an oral proteasome inhibitor) with lenalidomide and dexamethasone, and elotuzumab (a monoclonal antibody targeting SLAMF7) with lenalidomide and dexamethasone. Panobinostat received accelerated approval based on the PANORAMA1 trial, though its indication was later withdrawn in 2022 due to incomplete post-marketing data [16]. Ixazomib demonstrated improved median progression-free survival (PFS) in the TOURMALINE-MM1 trial (20.6 vs. 14.7 months; HR, 0.74; *P* = .012) [17]. Elotuzumab showed a 30% reduction in the risk of progression or death in the ELOQUENT-2 study (median PFS: 19.4 vs. 14.9 months; HR, 0.70; *P* = 0.0004) [18].

Daratumumab, an anti-CD38 monoclonal antibody, emerged as a pivotal agent during this interval. Initially approved in 2015 as monotherapy for heavily pretreated patients, its indications expanded rapidly. In 2016, the FDA approved daratumumab in combination with lenalidomide and dexamethasone (POLLUX trial) and with bortezomib and dexamethasone (CASTOR trial), both demonstrating significant PFS benefits [19, 20]. In POLLUX, median PFS was not reached in the daratumumab arm versus 18.4 months in the control group; in CASTOR, median PFS was not reached versus 7.2 months, respectively. In 2019, daratumumab was approved for newly diagnosed MM based on the MAIA trial, which showed a median PFS not reached in the daratumumab arm compared to 31.9 months in 2019, daratumumab was approved for newly diagnosed multiple myeloma based on the MAIA trial, which showed a median progression-free survival not reached in the control group, demonstrating substantial clinical benefit [21].

Additional therapeutic approvals between 2017 and 2021 further contributed to the evolving treatment landscape and likely reinforced the continued decline in MM-specific mortality. In 2017, lenalidomide was approved by the FDA as maintenance therapy for patients with newly diagnosed MM who had undergone autologous stem cell transplantation (ASCT). This decision was based on the CALGB 100104 trial, which demonstrated a significant improvement in time to progression (TTP: 57.3 vs. 28.9 months; HR, 0.57; 95% CI: 0.46–0.71; *P* < 0.0001) and overall survival [22].

In 2019, selinexor—a selective inhibitor of nuclear export and NF-κB signaling—was approved for triple-class refractory MM. In combination with dexamethasone, selinexor achieved a partial response or better in 26% of patients, with a median overall survival of 8.6 months and a median progression-free survival (PFS) of 3.7 months [23]. The following year, belantamab mafodotin-blmf became the first antibody-drug conjugate (ADC) approved for MM, based on the DREAMM-2 study, which demonstrated durable overall response rates in relapsed/refractory patients [24].

Also in 2020, isatuximab—an anti-CD38 monoclonal antibody—was approved in combination with pomalidomide and dexamethasone for relapsed/refractory MM. The ICARIA-MM trial showed improved PFS (11.53 vs. 6.47 months; HR, 0.596; 95% CI: 0.44–0.81; *P* = 0.0010) [25]. In 2021, isatuximab received expanded approval for use with carfilzomib and dexamethasone, based on the IKEMA trial, which reported a median PFS not reached in the isatuximab arm versus 20.27 months in the control arm [26].

These approvals reflect continued mechanistic diversification and therapeutic refinement in MM management. While individual agents varied in efficacy and durability, their collective integration into clinical practice likely contributed to sustained improvements in disease control and survival outcomes at the population level.

Between 2021 and 2023, the age-adjusted U.S. mortality rate for multiple myeloma (MM) declined sharply, representing the steepest drop observed across the entire study period. This trend coincided with the clinical uptake of advanced immunotherapies, including CAR T-cell therapies, bispecific antibodies, and optimized combination regimens. In 2021, the FDA approved idecabtagene vicleucel (ide-cel), the first anti-BCMA CAR T-cell therapy for relapsed/refractory MM, based on the Phase II KarMMa trial, which demonstrated unprecedented response rates in heavily pretreated patients [27]. In 2022, ciltacabtagene autoleucel (CARVYKTI) received FDA approval following the CARTITUDE-1 study, which reported an overall response rate of 98% and a stringent complete response rate of 78% [28]. That same year, teclistamab-cqyv—a bispecific BCMA-directed CD3 T-cell engager—was approved for patients with ≥4 prior lines of therapy, based on the MajesTEC-1 study showing an overall response rate of 61.8% [29].

These approvals reflect a new phase in MM management, defined by immune-based precision therapies capable of inducing deep and durable responses in heavily pretreated patients. While long-term survival data for CAR T-cell and bispecific antibody therapies are still maturing, their rapid clinical uptake and high response rates suggest meaningful contributions to the observed mortality decline. Importantly, these agents were introduced into a treatment landscape already shaped by decades of pharmacologic innovation, improved supportive care, and optimized sequencing strategies. The convergence of these factors—rather than any single intervention—likely accounts for the accelerated survival gains seen during this period. This reinforces the importance of continued investment in therapeutic diversity, real-world implementation, and longitudinal outcome tracking to sustain progress in MM care.

Recent studies underscore significant global and regional disparities in multiple myeloma (MM) trends. Globally, both age-standardized incidence rates (ASIR) and age-standardized mortality rates (ASMR) have increased over recent decades, with middle Socio-Demographic Index regions experiencing disproportionate impact [30, 31]. Europe reports the highest ASIR and prevalence rates, likely reflecting more widespread diagnostic capabilities and registry infrastructure [30]. However, mortality trends within Europe are heterogeneous—countries such as Sweden have reported declines, whereas others, including Bulgaria, continue to show rising death rates [31]. In North America, regional contrasts are also evident. While aggregate data suggest rising MM mortality across the continent [30], U.S.-specific analyses reveal a sustained decline, attributed to earlier detection, therapeutic innovation, and improved access to specialized care [32, 33]. These findings highlight the need for regionally tailored strategies and underscore the importance of global dissemination of advanced therapies to mitigate MM’s growing burden. Although national mortality has declined, access to novel therapies remains uneven. Geographic, socioeconomic, and insurance-related barriers may limit who benefits from recent advances. Expanding referral pathways, reducing financial barriers, and improving access to specialized centers will be essential to ensure these survival gains are equitably realized.

Recent clinical trials have demonstrated that achieving minimal residual disease (MRD) negativity is strongly associated with prolonged progression-free and overall survival in multiple myeloma. Importantly, MRD negativity is now achievable in a substantial proportion of patients with both newly diagnosed and relapsed disease, reflecting the increasing depth of response attainable with modern therapies. These developments underscore the need for repeated population-level and patient-level analyses, as the rapid pace of therapeutic innovation continues to shift response benchmarks and long-term outcomes.

Recent therapeutic combinations, including the integration of CD38 monoclonal antibodies with lenalidomide-, bortezomib-, and dexamethasone-based regimens in the context of high-dose melphalan and autologous stem cell transplantation, have produced unprecedented depth and durability of response in newly diagnosed multiple myeloma. These advances further reinforce the need for ongoing population-level analyses as evolving combination strategies continue to shift long-term outcome expectations.

Similar declines in cancer-specific mortality have been observed in other malignancies, including lung cancer and melanoma [33, 34]. While these trends reflect meaningful progress, they also underscore the rising cost of cancer care [35]. As survival improves in multiple myeloma and other cancers, long-term toxicity management becomes a clinical priority. Prolonged exposure to novel therapies demands recognition and treatment of rare or delayed toxicities [36, 37]. Emerging biomarkers and sequencing tools increasingly enable prediction of response and toxicity risk, guiding personalized care [38–40]. Contemporary MM therapies—particularly those used in maintenance settings or across multiple lines—are associated with cumulative side effects, including peripheral neuropathy, cytopenias, cardiovascular risks, and immune dysfunction. Addressing these challenges will require multidisciplinary survivorship models and equitable access to supportive care services to preserve quality of life.

The financial burden of MM treatment continues to escalate, especially with the introduction of high-cost therapies such as CAR T-cell products and bispecific antibodies. Recent analyses highlight cost-effectiveness concerns and reinforce the need for optimized treatment sequencing, value-based care models, and policy-level interventions to ensure sustainable access to life-extending therapies [41].

Looking ahead, the therapeutic landscape of MM is rapidly evolving toward rational combination strategies that integrate immunotherapies, targeted agents, and established backbone regimens. Early-phase data suggest that combining bispecific antibodies with IMiDs or proteasome inhibitors may enhance depth and durability of response, even in heavily pretreated populations. Similarly, trials are exploring the sequential or concurrent use of CAR T-cell therapies with checkpoint inhibitors or gamma-secretase modulators to overcome resistance and improve persistence.

Beyond efficacy, future combinations aim to reduce cumulative toxicity and improve tolerability—particularly for older adults and patients with comorbidities. As MM management becomes increasingly personalized, treatment selection will likely be guided by cytogenetic risk, minimal residual disease (MRD) status, and immune profiling. Continued innovation in trial design, biomarker development, and real-world data integration will be essential to refine the role of combination therapy in achieving sustained remission and improving long-term survival outcomes.

### Strengths and limitations

A key strength of this study lies in its use of a large, population-based sample derived from the publicly available SEER database, which is broadly representative of the U.S. population. Unlike clinical trials that often exclude older adults or socioeconomically disadvantaged groups, SEER captures real-world data across diverse demographics, enhancing the generalizability of our findings. The extended study period (1975–2023) allows for longitudinal analysis of MM-specific mortality trends across multiple therapeutic eras, including the most recent approvals of CAR T-cell and bispecific antibody therapies. By focusing on age-adjusted mortality rates, the study provides a standardized and clinically meaningful metric for assessing population-level impact.

However, several limitations must be acknowledged. As a retrospective observational analysis, the study is subject to unmeasured confounding and selection bias inherent to registry-based datasets. The SEER database lacks granular clinical information, including cytogenetic risk, minimal residual disease (MRD) status, comorbidities, and specific treatment regimens, which limits mechanistic interpretation. Variability in treatment protocols, adherence, and reporting practices across institutions may also introduce heterogeneity. Finally, while temporal associations between therapeutic approvals and mortality trends are compelling, they do not establish causality.

Despite these limitations, our findings offer critical insights into the real-world impact of evolving MM treatment strategies. The use of the most recent SEER data through 2023 provides a timely snapshot of survival outcomes in the context of rapidly advancing therapeutic options. As the MM survivor population grows, future research must address survivorship care, long-term toxicity management, and the financial sustainability of high-cost therapies to ensure equitable benefit across patient populations.

## MATERIALS AND METHODS

### Data source

This retrospective population-based study utilized publicly available U.S. mortality data from the Surveillance, Epidemiology, and End Results (SEER) Program, curated by the National Cancer Institute (NCI). Mortality statistics were accessed via SEER*Explorer (https://seer.cancer.gov/statistics-network/explorer/, accessed on 24 June 2025), derived from the U.S. Mortality Files (1969–2023) compiled by the National Center for Health Statistics and Centers for Disease Control and Prevention [42, 43]. Multiple myeloma–specific mortality was identified using the SEER Cause of Death Recode (1969+), with all rates age-adjusted to the 2000 U.S. Standard Population (Census P25-1130, 20 age groups). Data access was granted under an official SEER Data Use Agreement. Ethical approval was not required due to the use of publicly available, de-identified data.

### Study population and case definition

The study population comprised U.S. adults aged 18 years or older who died from multiple myeloma (International Classification of Diseases for Oncology, Third Edition (ICD-O-3) code 9732/3) between January 1, 1975 and December 31, 2023. Individuals with missing age or cause-of-death data were excluded. Only malignant cases with confirmed myeloma as the underlying cause of death were included. The analysis encompassed all races and both sexes.

### Temporal contextualization

To aid interpretation of observed mortality trends, calendar years were grouped into six intervals that broadly reflect shifts in clinical practice and therapeutic availability. These intervals are not intended to represent discrete or causally defined “eras,” but rather to provide clinical context for evaluating population-level changes over time. Treatment advances—including stem cell transplantation, proteasome inhibitors, immunomodulatory drugs, monoclonal antibodies, and cellular immunotherapies—were introduced gradually and often overlapped. As such, observed mortality shifts likely reflect a cumulative and multifactorial impact of evolving treatment strategies, supportive care improvements, and broader health system dynamics.

### Statistical analysis

Age-adjusted mortality rates per 100,000 population were obtained for each calendar year using SEER*Explorer. Trend estimates were derived from SEER’s built-in analytic framework, which applies Joinpoint regression modeling to identify statistically significant changes in mortality over time. Annual Percent Change (APC) and 95% confidence intervals (CIs) were reported for each segment. A trend was considered increasing if the CI was entirely above 0, decreasing if entirely below 0, and not statistically significant if the CI spanned 0. Statistical significance was defined as *P* < .05.

### Reporting guidelines

This study adheres to the Strengthening the Reporting of Observational Studies in Epidemiology (STROBE) guidelines, including the recommendations outlined in the STROBE Explanation and Elaboration document. The manuscript fulfills STROBE criteria by clearly defining the study design, data sources, study population, variables, statistical methods, and limitations, and by transparently reporting results in a structured and reproducible manner [44].

## CONCLUSIONS

The declining MM-specific mortality rates over the past five decades underscore the transformative impact of therapeutic innovation on patient survival. The introduction of novel drug classes, intensified frontline regimens, and optimized sequencing strategies has contributed to substantial improvements, reinforcing MM’s transition into a more manageable chronic condition.

While these advancements have reshaped MM treatment paradigms, they also introduce new challenges—including long-term toxicities, disparities in healthcare access, and escalating financial burdens. Addressing these issues will be essential to ensure that the benefits of innovation are equitably distributed across diverse patient populations.

Looking ahead, the durability and depth of response associated with emerging therapies—particularly CAR T-cell products and bispecific antibodies—suggest the potential for even more pronounced declines in MM-specific mortality. As these agents move earlier in the treatment algorithm and are integrated into combination strategies, their population-level impact may accelerate. Our study, leveraging the most recent SEER data through 2023, offers a timely snapshot of this evolving landscape and provides a foundation for future research into survivorship, affordability, and global dissemination of advanced MM therapies.

## References

[1] van de Donk NWC, Pawlyn C, Yong KL. Multiple myeloma. Lancet. 2021; 397:410–27. 10.1016/S0140-6736(21)00135-5. 33516340

[2] Attal M, Harousseau JL, Stoppa AM, Sotto JJ, Fuzibet JG, Rossi JF, Casassus P, Maisonneuve H, Facon T, Ifrah N, Payen C, Bataille R. A prospective, randomized trial of autologous bone marrow transplantation and chemotherapy in multiple myeloma. Intergroupe Français du Myélome. N Engl J Med. 1996; 335:91–97. 10.1056/NEJM199607113350204. 8649495

[3] Ito S. Proteasome Inhibitors for the Treatment of Multiple Myeloma. Cancers (Basel). 2020; 12:265. 10.3390/cancers12020265. 31979059 PMC7072336

[4] Holstein SA, McCarthy PL. Immunomodulatory Drugs in Multiple Myeloma: Mechanisms of Action and Clinical Experience. Drugs. 2017; 77:505–20. 10.1007/s40265-017-0689-1. 28205024 PMC5705939

[5] D’Agostino M, Innorcia S, Boccadoro M, Bringhen S. Monoclonal Antibodies to Treat Multiple Myeloma: A Dream Come True. Int J Mol Sci. 2020; 21:8192. 10.3390/ijms21218192. 33139668 PMC7662679

[6] Durie BGM, Hoering A, Rajkumar SV, Abidi MH, Epstein J, Kahanic SP, Thakuri MC, Reu FJ, Reynolds CM, Sexton R, Orlowski RZ, Barlogie B, Dispenzieri A. Bortezomib, lenalidomide and dexamethasone versus lenalidomide and dexamethasone in patients with previously untreated multiple myeloma without intent for immediate autologous stem cell transplant (SWOG S0777): results of the randomized phase III trial. Blood. 2015; 126:25. 10.1182/blood.V126.23.25.25.

[7] Siegel RL, Miller KD, Fuchs HE, Jemal A. Cancer statistics, 2022. CA Cancer J Clin. 2022; 72:7–33. 10.3322/caac.21708. 35020204

[8] Kyle RA, Rajkumar SV. Treatment of multiple myeloma. Blood. 2004; 103:20–32. 10.1182/blood-2003-05-1635. 12969978

[9] Singhal S, Mehta J, Desikan R, Ayers D, Roberson P, Eddlemon P, Munshi N, Anaissie E, Wilson C, Dhodapkar M, Zeddis J, Barlogie B. Antitumor activity of thalidomide in refractory multiple myeloma. N Engl J Med. 1999; 341:1565–71. 10.1056/NEJM199911183412102. 10564685

[10] Barlogie B, Desikan R, Eddlemon P, Spencer T, Zeldis J, Munshi N, Badros A, Zangari M, Anaissie E, Epstein J, Shaughnessy J, Ayers D, Spoon D, Tricot G. Extended survival in advanced and refractory multiple myeloma after single-agent thalidomide: identification of prognostic factors in a phase 2 study of 169 patients. Blood. 2001; 98:492–94. 10.1182/blood.v98.2.492. 11435324

[11] Rajkumar SV, Hayman SR, Gertz MA. Combination therapy with thalidomide plus dexamethasone in newly diagnosed multiple myeloma: a randomized trial. J Clin Oncol. 2006; 24:431–436.

[12] Ning YM, He K, Dagher R, Sridhara R, Farrell AT, Justice R, Pazdur R. Liposomal doxorubicin in combination with bortezomib for relapsed or refractory multiple myeloma. Oncology (Williston Park). 2007; 21:1503–8. 18077994

[13] San Miguel JF, Schlag R, Khuageva NK, Dimopoulos MA, Shpilberg O, Kropff M, Spicka I, Petrucci MT, Palumbo A, Samoilova OS, Dmoszynska A, Abdulkadyrov KM, Schots R, et al, and VISTA Trial Investigators. Bortezomib plus melphalan and prednisone for initial treatment of multiple myeloma. N Engl J Med. 2008; 359:906–17. 10.1056/NEJMoa0801479. 18753647

[14] Siegel DS, Martin T, Wang M, Vij R, Jakubowiak AJ, Lonial S, Trudel S, Kukreti V, Bahlis N, Alsina M, Chanan-Khan A, Buadi F, Reu FJ, et al. A phase 2 study of single-agent carfilzomib (PX-171-003-A1) in patients with relapsed and refractory multiple myeloma. Blood. 2012; 120:2817–25. 10.1182/blood-2012-05-425934. 22833546 PMC4123387

[15] Miguel JS, Weisel K, Moreau P, Lacy M, Song K, Delforge M, Karlin L, Goldschmidt H, Banos A, Oriol A, Alegre A, Chen C, Cavo M, et al. Pomalidomide plus low-dose dexamethasone versus high-dose dexamethasone alone for patients with relapsed and refractory multiple myeloma (MM-003): a randomised, open-label, phase 3 trial. Lancet Oncol. 2013; 14:1055–66. 10.1016/S1470-2045(13)70380-2. 24007748

[16] San-Miguel JF, Hungria VT, Yoon SS, Beksac M, Dimopoulos MA, Elghandour A, Jedrzejczak WW, Günther A, Nakorn TN, Siritanaratkul N, Corradini P, Chuncharunee S, Lee JJ, et al. Panobinostat plus bortezomib and dexamethasone versus placebo plus bortezomib and dexamethasone in patients with relapsed or relapsed and refractory multiple myeloma: a multicentre, randomised, double-blind phase 3 trial. Lancet Oncol. 2014; 15:1195–206. 10.1016/S1470-2045(14)70440-1. 25242045

[17] Moreau P, Masszi T, Grzasko N, Bahlis NJ, Hansson M, Pour L, Sandhu I, Ganly P, Baker BW, Jackson SR, Stoppa AM, Simpson DR, Gimsing P, et al, and TOURMALINE-MM1 Study Group. Oral Ixazomib, Lenalidomide, and Dexamethasone for Multiple Myeloma. N Engl J Med. 2016; 374:1621–34. 10.1056/NEJMoa1516282. 27119237

[18] Lonial S, Dimopoulos M, Palumbo A, White D, Grosicki S, Spicka I, Walter-Croneck A, Moreau P, Mateos MV, Magen H, Belch A, Reece D, Beksac M, et al, and ELOQUENT-2 Investigators. Elotuzumab Therapy for Relapsed or Refractory Multiple Myeloma. N Engl J Med. 2015; 373:621–31. 10.1056/NEJMoa1505654. 26035255

[19] Palumbo A, Chanan-Khan A, Weisel K, Nooka AK, Masszi T, Beksac M, Spicka I, Hungria V, Munder M, Mateos MV, Mark TM, Qi M, Schecter J, et al, and CASTOR Investigators. Daratumumab, Bortezomib, and Dexamethasone for Multiple Myeloma. N Engl J Med. 2016; 375:754–66. 10.1056/NEJMoa1606038. 27557302

[20] Dimopoulos MA, Oriol A, Nahi H, San-Miguel J, Bahlis NJ, Usmani SZ, Rabin N, Orlowski RZ, Komarnicki M, Suzuki K, Plesner T, Yoon SS, Ben Yehuda D, et al, and POLLUX Investigators. Daratumumab, Lenalidomide, and Dexamethasone for Multiple Myeloma. N Engl J Med. 2016; 375:1319–31. 10.1056/NEJMoa1607751. 27705267

[21] Facon T, Kumar S, Plesner T, Orlowski RZ, Moreau P, Bahlis N, Basu S, Nahi H, Hulin C, Quach H, Goldschmidt H, O’Dwyer M, Perrot A, et al, and MAIA Trial Investigators. Daratumumab plus Lenalidomide and Dexamethasone for Untreated Myeloma. N Engl J Med. 2019; 380:2104–15. 10.1056/NEJMoa1817249. 31141632 PMC10045721

[22] McCarthy PL, Holstein SA, Petrucci MT, Richardson PG, Hulin C, Tosi P, Bringhen S, Musto P, Anderson KC, Caillot D, Gay F, Moreau P, Marit G, et al. Lenalidomide Maintenance After Autologous Stem-Cell Transplantation in Newly Diagnosed Multiple Myeloma: A Meta-Analysis. J Clin Oncol. 2017; 35:3279–89. 10.1200/JCO.2017.72.6679. 28742454 PMC5652871

[23] Chari A, Vogl DT, Gavriatopoulou M, Nooka AK, Yee AJ, Huff CA, Moreau P, Dingli D, Cole C, Lonial S, Dimopoulos M, Stewart AK, Richter J, et al. Oral Selinexor-Dexamethasone for Triple-Class Refractory Multiple Myeloma. N Engl J Med. 2019; 381:727–38. 10.1056/NEJMoa1903455. 31433920

[24] Lonial S, Lee HC, Badros A, Trudel S, Nooka AK, Chari A, Abdallah AO, Callander N, Lendvai N, Sborov D, Suvannasankha A, Weisel K, Karlin L, et al. Belantamab mafodotin for relapsed or refractory multiple myeloma (DREAMM-2): a two-arm, randomised, open-label, phase 2 study. Lancet Oncol. 2020; 21:207–21. 10.1016/S1470-2045(19)30788-0. 31859245

[25] Attal M, Richardson PG, Rajkumar SV, San-Miguel J, Beksac M, Spicka I, Leleu X, Schjesvold F, Moreau P, Dimopoulos MA, Huang JS, Minarik J, Cavo M, et al, and ICARIA-MM study group. Isatuximab plus pomalidomide and low-dose dexamethasone versus pomalidomide and low-dose dexamethasone in patients with relapsed and refractory multiple myeloma (ICARIA-MM): a randomised, multicentre, open-label, phase 3 study. Lancet. 2019; 394:2096–107. 10.1016/S0140-6736(19)32556-5. 31735560

[26] Moreau P, Dimopoulos MA, Mikhael J, Yong K, Capra M, Facon T, Hajek R, Špička I, Baker R, Kim K, Martinez G, Min CK, Pour L, et al, and IKEMA study group. Isatuximab, carfilzomib, and dexamethasone in relapsed multiple myeloma (IKEMA): a multicentre, open-label, randomised phase 3 trial. Lancet. 2021; 397:2361–71. 10.1016/S0140-6736(21)00592-4. 34097854

[27] Munshi NC, Anderson LD Jr, Shah N, Madduri D, Berdeja J, Lonial S, Raje N, Lin Y, Siegel D, Oriol A, Moreau P, Yakoub-Agha I, Delforge M, et al. Idecabtagene Vicleucel in Relapsed and Refractory Multiple Myeloma. N Engl J Med. 2021; 384:705–16. 10.1056/NEJMoa2024850. 33626253

[28] Berdeja JG, Madduri D, Usmani SZ, Jakubowiak A, Agha M, Cohen AD, Stewart AK, Hari P, Htut M, Lesokhin A, Deol A, Munshi NC, O’Donnell E, et al. Ciltacabtagene autoleucel, a B-cell maturation antigen-directed chimeric antigen receptor T-cell therapy in patients with relapsed or refractory multiple myeloma (CARTITUDE-1): a phase 1b/2 open-label study. Lancet. 2021; 398:314–24. 10.1016/S0140-6736(21)00933-8. 34175021

[29] Moreau P, Garfall AL, van de Donk NWC, Nahi H, San-Miguel JF, Oriol A, Nooka AK, Martin T, Rosinol L, Chari A, Karlin L, Benboubker L, Mateos MV, et al. Teclistamab in Relapsed or Refractory Multiple Myeloma. N Engl J Med. 2022; 387:495–505. 10.1056/NEJMoa2203478. 35661166 PMC10587778

[30] Global Burden of Disease Collaborative Network. Global burden of multiple myeloma: incidence, mortality, and DALYs, 1990–2021. BMC Public Health. 2025; 25:22240. 10.1186/s12889-025-22240-2.

[31] Sun J, Li X, Chen Q. Regional disparities in global multiple myeloma burden: incidence, mortality, and DALY trends from 1990 to 2021. Lancet Haematol. 2023; 8:e798–810.

[32] Li T, Sun X, Wang D. Trends in multiple myeloma incidence and mortality in the USA (1999–2020): a SEER database and CDC WONDER analysis. Sci Rep. 2024; 14:65590.

[33] Kahlon N, Doddi S, Yousif R, Najib S, Sheikh T, Abuhelwa Z, Burmeister C, Hamouda DM. Melanoma Treatments and Mortality Rate Trends in the US, 1975 to 2019. JAMA Netw Open. 2022; 5:e2245269. 10.1001/jamanetworkopen.2022.45269. 36472871 PMC9856246

[34] Howlader N, Forjaz G, Mooradian MJ, Meza R, Kong CY, Cronin KA, Mariotto AB, Lowy DR, Feuer EJ. The Effect of Advances in Lung-Cancer Treatment on Population Mortality. N Engl J Med. 2020; 383:640–49. 10.1056/NEJMoa1916623. 32786189 PMC8577315

[35] Chen S, Cao Z, Prettner K, Kuhn M, Yang J, Jiao L, Wang Z, Li W, Geldsetzer P, Bärnighausen T, Bloom DE, Wang C. Estimates and Projections of the Global Economic Cost of 29 Cancers in 204 Countries and Territories From 2020 to 2050. JAMA Oncol. 2023; 9:465–72. 10.1001/jamaoncol.2022.7826. 36821107 PMC9951101

[36] Kahlon N, Vallepu S, Manje Gowda A, Baddam S. Pemetrexed-induced lipodermatosclerosis: clinical course and management of a rare cutaneous toxicity. Curr Probl Cancer Case Rep. 2025; 19:100386. 10.1016/j.cpccr.2025.100386.

[37] Baddam S, Patel S, Kahlon N, Thiriveedi M. Unmasking Myelin Oligodendrocyte Glycoprotein Antibody-Associated Disease (MOGAD): CNS Demyelination Triggered by TNF-α Inhibition in a Patient with Ankylosing Spondylitis. Eur J Case Rep Intern Med. 2025; 12:005467. 10.12890/2025_005467. 40502939 PMC12151557

[38] Qureshi Z, Jamil A, Hameed F, Kahlon N. Advancements in Cutaneous T-Cell Lymphoma Treatment: Unveiling Novel Therapeutic Avenues and Clinical Implications. Am J Clin Oncol. 2025; 48:623–28. 10.1097/COC.0000000000001230. 40637334

[39] Kahlon N, Baddam S, Bansal N, Qureshi Z, Maradana J. Dynamic Changes in Breast Cancer Receptor Status: A Case Report Highlighting the Importance of Repeat Biopsies in Guiding Treatment Strategies. J Investig Med High Impact Case Rep. 2025; 13:23247096251362974. 10.1177/23247096251362974. 40727978 PMC12314236

[40] Verghese C, Li W, Gvazava N, Alimpertis E, Kahlon N, Sun H, Booth R. IGH/BCL2 Status Better Predicts Clinico-Pathological Behavior in Primary Splenic Follicular Lymphoma than Histological Grade and Other Molecular Markers. Clin Pathol. 2022; 15:2632010X221129242. 10.1177/2632010X221129242. 36313587 PMC9608027

[41] Keesari PR, Samuels D, Vegivinti CTR, Pulakurthi YS, Kudithi R, Dhar M, Janakiram M. Navigating the Economic Burden of Multiple Myeloma: Insights into Cost-effectiveness of CAR-T and Bispecific Antibody Therapies. Curr Hematol Malig Rep. 2025; 20:3. 10.1007/s11899-024-00748-5. 39754658 PMC11700040

[42] Surveillance Research Program, National Cancer Institute. SEER*Explorer: An interactive website for SEER cancer statistics. 2024. https://seer.cancer.gov/statistics-network/explorer/.

[43] Statistical Methodology and Applications Branch, Surveillance Research Program, National Cancer Institute. Joinpoint Regression Program, Version 5.1. 2024. https://surveillance.cancer.gov/joinpoint/.

[44] Vandenbroucke JP, von Elm E, Altman DG, Gøtzsche PC, Mulrow CD, Pocock SJ, Poole C, Schlesselman JJ, Egger M, and STROBE initiative. Strengthening the Reporting of Observational Studies in Epidemiology (STROBE): explanation and elaboration. Ann Intern Med. 2007; 147:W163–94. 10.7326/0003-4819-147-8-200710160-00010-w1. 17938389

